# Acute Exercise Effects on Cognitive Flexibility in Preterm and Full-Term Children: An Event-Related Potential Study

**DOI:** 10.7150/ijms.120694

**Published:** 2026-01-01

**Authors:** Feng-Tzu Chen, Pei-Chen Hsiao, Charles H. Hillman, Sheng-Hsien Feng, Chien-Heng Chu, Yu-Kai Chang

**Affiliations:** 1 Department of Kinesiology, National Tsing Hua University, Hsinchu, Taiwan.; 2 Research Center for Education and Mind Sciences, National Tsing Hua University, Hsinchu, Taiwan.; 3 Department of Physical Education and Sports Sciences, National Taiwan Normal University, Taipei, Taiwan.; 4 Department of Psychology, Northeastern University, Boston, Massachusetts, USA.; 5 Department of Physical Therapy, Movement, and Rehabilitation Sciences, Northeastern University, Boston, Massachusetts, USA.; 6 Institute for Cognitive and Brain Health, Northeastern University, Boston, Massachusetts, USA.; 7 The National Shooting Training Base Gongxi Shooting Range, National Sports Training Center, Taoyuan, Taiwan.; 8 Institute of Sport Science and Innovations, Lithuanian Sports University, Kaunas, Lithuania.

**Keywords:** acute exercise, executive function, cognitive function, preterm, children

## Abstract

**Background:** Preterm birth is associated with impairments in executive functions (EFs), particularly in cognitive flexibility, which is essential for adaptive and goal-directed behavior. While acute exercise has been shown to transiently enhance cognitive flexibility in children born full-term, its effects in preterm children remain poorly understood. This study aimed to examine the effects of acute aerobic exercise on cognitive flexibility and its underlying neural mechanisms in preterm children, and to determine whether these effects are comparable to those observed in full-term peers.

**Methods:** Children aged between 10 and 16 years were assigned based on gestational age to either the preterm group (n = 20; born before 37 weeks of gestation) or the full-term group (n = 22; born at or after 37 weeks) to complete two sessions, including a 30-minute aerobic exercise (AE) session and a seated control (CON) session. Cognitive flexibility was assessed immediately after each session using a task-switching paradigm, with concurrent electroencephalographic recording to measure P3b event-related potentials (ERPs).

**Results:** Across both groups, participants exhibited shorter response times in the global and local switch conditions and higher accuracy in the local switch condition following AE compared with CON, although switching costs did not differ significantly between sessions. ERP analyses showed increased P3b amplitudes after AE in both switch conditions, indicating enhanced allocation of attentional resources. No significant group differences were observed, suggesting comparable behavioral and neural patterns between preterm and full-term children.

**Conclusion:** These findings indicate that a single session of moderate-intensity aerobic exercise may transiently enhance cognitive processing in both preterm and full-term children. Although behavioral improvements were not observed in the core index of cognitive flexibility (i.e., switching cost), the ERP results suggest a short-term modulation of neural efficiency following acute exercise.

## 1. Background

Preterm birth, defined as birth at a gestational age of less than 37 weeks, accounted for approximately 9.9% of live births globally in 2020, equating to 13.4 million cases 1. Beyond the well-documented risks of cerebral palsy, intellectual disabilities, hearing and visual impairments, there is growing recognition that children born preterm are also vulnerable to neurodevelopmental difficulties, including academic underachievement 2, behavioral problems 3, and deficits in executive functions (EFs) 4, 5, which persist from childhood to young adulthood. Evidence has shown that academic underachievement and behavioral problems in childhood arise from deficits in EFs 6, a set of higher-order cognitive processes essential for regulating top-down, goal-directed behaviors, and consisting of three core components including inhibitory control, working memory, and cognitive flexibility 7, 8. Of these three EFs, cognitive flexibility exhibits the most pronounced deficits in individuals with a history of preterm birth compared to those born full-term 9. Consequently, considerable attention has been paid to identifying effective interventions to enhance the cognitive flexibility of preterm children.

Among the various interventions aimed at enhancing EFs in children, acute exercise (also known as a single bout of exercise) has gained increasing attention for its immediate cognitive benefits 10. Previous meta-analytic reviews have demonstrated that acute exercise elicits small to moderate enhancements in EF performance among healthy individuals 11-13. More recently, an umbrella review synthesizing findings across 30 meta-analyses reported that these benefits are particularly pronounced in children and adolescents, with the greatest effects observed in those under 18 years of age (*ES* = 0.33), compared to smaller effects in other age groups [i.e., young, middle-aged, and older adults; 14]. These findings underscore the developmental sensitivity of cognitive functions during childhood and adolescence, suggesting a heightened responsiveness of the physiological system to stimulation induced by a single bout of exercise.

While the transient cognitive benefits of acute exercise are well-documented in typically developing populations (i.e., full-term individuals), evidence regarding its efficacy in children with neurodevelopmental conditions remains limited. Nonetheless, previous studies have linked acute aerobic exercise to improvements in cognitive flexibility among children with conditions such as attention-deficit/hyperactivity disorder [ADHD; 11, 15, 16]. Recently, two studies conducted by our research group have also indicated that children born preterm may benefit from acute exercise. Specifically, Chen, Feng 17 reported that a single session of moderate-intensity aerobic exercise significantly enhanced inhibitory control, as measured by the numerical Stroop task, compared to a reading control session in children born preterm. Similarly, Ren, Feng 18 found that both aerobic and resistance-based acute exercise improved planning performance, as assessed by the Tower of London task, in this population. Despite these preliminary findings, no studies to date have examined whether the magnitude of EF improvements following acute exercise differs between children born preterm and their full-term peers. Moreover, prior research has primarily focused on inhibition and planning, with relatively limited attention given to cognitive flexibility—arguably the most compromised EF domain in individuals born preterm. Accordingly, further investigation is warranted to determine whether acute exercise can elicit comparable or differential benefits in cognitive flexibility among preterm and full-term children.

As improvements in EFs after acute moderate-intensity exercise have been linked to optimal facilitation of neurocognitive function, event-related potentials (ERPs) may contribute to a deeper understanding of the underlying mechanisms. The high temporal resolution of ERPs from electroencephalography (EEG) offers a comprehensive means of assessing mental processing during task stimuli that modulate EF demands. P3b (also referred to as P3 or P300), a late endogenous component with a topographic maximum over parietal electrode sites (e.g., Pz), is a neuroelectric marker used to reflect aspects of information processing involved in EFs, and is most commonly applied in studies that address acute exercise and neurocognitive function 19. Previous ERP studies demonstrated superior cognitive flexibility performance following acute aerobic exercise using the task-switching paradigm and simultaneously showed larger P3b amplitude, suggesting that acute exercise improves EFs via enhancement of attentional resource allocation 16, 20. However, to date, no studies have specifically examined whether acute exercise modulates P3b in preterm children, revealing a significant research gap in the neuroelectric effects of acute exercise in this population.

The present study investigated the effects of acute aerobic exercise on EFs in preterm children relative to full-term peers, integrating behavioral and neuroelectric assessments. Specifically, we examined how a single session of moderate-intensity exercise influenced cognitive flexibility and the P3b component of a stimulus-locked ERP. Two primary hypotheses were proposed. First, cognitive flexibility performance was expected to be higher following the aerobic exercise (AE) session compared with the control (CON) session, with no significant differences between preterm and full-term children. Second, P3b amplitude was expected to be larger after the AE session than after the CON session, again without significant group differences. These hypotheses were formulated to determine whether preterm children experience cognitive and neurophysiological benefits from acute exercise comparable to those observed in their full-term peers.

## 2. Methods

### 2.1. Participants

Fifty children, 25 born preterm and 25 born full-term, were recruited through advertisements distributed to local elementary schools in Taipei City. An a priori power analysis was conducted using parameters (α = .05, power = .80, partial η² = .18) obtained from a comparable study 17 to determine the minimum sample size required. The participants were categorized into the preterm or full-term groups based on gestational age of < 37 weeks for preterm and 37 ≤ weeks for full-term. The eligibility criteria included the following: (1) age between 10 and 16 years; (2) no history of brain injury, psychological disorders, or neurological diseases; (3) no use of medications that could influence neurocognitive function; (4) absence of comorbid developmental disorders (e.g., learning disabilities); and (5) no health risks that would contraindicate participation in a 20-minute bout of moderate-intensity exercise, as determined by the Physical Activity Readiness Questionnaire (PAR-Q). Neuroelectric data from five children in the preterm group and three in the full-term group were excluded due to poor signal quality. The final sample and demographic characteristics are summarized in **Table 1**.

### 2.2. Cardiorespiratory fitness assessment

Children's cardiorespiratory fitness was assessed using the YMCA submaximal cycling test 21 on an ergometer equipped with an electromagnetic braking system (Corival CPET, Lode, the Netherlands). The test consisted of consecutive 3-minute stages, beginning with an initial workload of 150 kg·m/min (equivalent to 25 W at 50 revolutions per minute [rpm]). Subsequent workloads were determined based on the heart rate (HR) measured at the second and third minutes of the first stage. The test was terminated when the HR at two consecutive stages fell within the range of 110 bpm to 85% of the participant's age-predicted maximum heart rate (HR_max_), calculated using the formula, HRmax = 206.9 - (0.67 × age) 22. Cardiorespiratory fitness was indexed by estimated maximal oxygen uptake (VO₂_max_), which was calculated using the standard YMCA prediction equation.

### 2.3. Intervention

Two intervention sessions, AE and CON, were implemented in a randomized crossover design. In the AE session, participants performed 30 minutes of cycling exercise on a cycle ergometer (Corival CPET), consisting of a 5-minute warm-up at 70 rpm, a 20-minute bout at moderate intensity (40-59% heart rate reserve, HRR), and a 5-minute cool-down. HRR was calculated as HRR = [(HRmax - resting HR) × target intensity (%)] + resting HR 23. n the CON session, participants read a picture book quietly for 30 minutes. Heart rate was continuously monitored every minute using a heart rate sensor (H10; Polar Electro Oy, Kempele, Finland) during both sessions.

### 2.4. Task-switching task

The task-switching task, programmed with NeuroScan STIM2 software (Neuro Inc., El Paso, TX, USA), was used to assess cognitive flexibility 20. The test consisted of 384 trials grouped into six blocks of 64 digital stimuli (digits 1 to 9, excluding 5), sequentially presented with a duration of 200 ms and a fixed intertrial interval of 3000 ms. Each stimulus was 2.3 cm tall and presented on a 15-inch LCD screen against a black background, with a viewing distance of approximately 65 cm. In Block One, digit stimuli were superimposed with solid-line rectangular boxes (e.g., AAAA...), and participants were instructed to identify whether the stimuli were larger or smaller than 5 (the high/low rule). In Block Two, digit stimuli were superimposed with dashed-line rectangular boxes (e.g., BBBB...), and participants were instructed to classify whether the stimuli were even or odd (the odd/even rule). During Blocks Three to Six, both types of digit stimuli appeared in alternating runs, each with run lengths of two (e.g., AABBAA...). Thus, trials in blocks one and two were homogeneous conditions, with a single task rule for each block, while trials in the last four blocks were heterogeneous conditions, with two possible task rules in each block. Furthermore, based on whether the current trial inherited the same or a different task rule from the preceding trial, the heterogeneous trials were further categorized into a non-switch condition (e.g., AA or BB) or a switch condition (e.g., AB or BA). Accordingly, there were 32 non-switch and 32 switch trials in each heterogeneous block. The alternating runs of the non-switch and switch trials ensured that the arousal levels and working memory demands across switch and non-switch trials were similar. Responses made outside the response window (i.e., beyond 2000 ms from the onset of the stimulus), as well as incorrect or omitted responses, were considered errors. The response time (RT) of correct responses and accuracy under homogeneous, heterogeneous, non-switch, and switch conditions were calculated as the behavioral indices. Additionally, the global switch RT cost (i.e., heterogeneous condition - homogeneous condition) and the local switch RT cost (i.e., switch trials - non-switch trials in the heterogeneous condition), as well as the global switch accuracy cost (i.e., homogeneous condition - heterogeneous condition) and the local switch accuracy cost (i.e., non-switch trials - switch trials in the heterogeneous condition) were calculated.

### 2.5. Neuroelectric recording and processing

Electroencephalography was recorded with 32 Ag/AgCl electrodes mounted on an elastic cap (Quik-Cap, NeuroScan Inc.) in accordance with the 10-20 standard 24, 25 using CURRY 8 Data Acquisition and Online Processing software (Compumedics Neuroscan, Charlotte, NC, USA). The electrode impedance of all electrodes was below 10 kΩ prior to and throughout the experiment. Additional electrodes were placed below and above the left eye, and the outer canthus of both eyes to monitor the electrooculograms. Continuous EEG was digitized at 1000 Hz, amplified 500×, and filtered at 60 Hz using a SynAmps2 amplifier (NeuroScan Inc.). Offline EEG processing consisted of the following steps. Trials with correct responses underwent ocular correction 26 and segmentation into 1200-ms epochs (-200 to 1000 ms relative to stimulus onset). Epochs were baseline corrected to the -100 to 0 ms pre-stimulus interval and filtered at 30 Hz (12 dB/octave). Epochs exceeding ±100 μV were rejected. Retained homogeneous trials during AE and CON for full-term participants had means of 121.13 (SD = 8.16) and 122.85 (SD = 7.91) trials, respectively, and for preterm participants had means of 123.36 (SD = 4.88) and 117.73 (SD = 22.02) trials, respectively. Retained non-switch trials during AE and CON for full-term participants had means of 126.12 (SD = 5.09) and 129.15 (SD = 4.55) trials, respectively, and for preterm participants had means of 128.09 (SD = 5.56) and 129.00 (SD = 4.10) trials, respectively. Retained switch trials during AE and CON for full-term participants had means of 121.33 (SD = 10.38) and 125.85 (SD = 9.49) trials, respectively, and for preterm participants had means of 126.27 (SD = 6.00) and 125.91 (SD = 10.33) trials, respectively.

After a preliminary visual inspection of the grand ERP average, P3b of each trial was defined as parietally distributed positivity from 430 to 630 ms. Average mean amplitudes were quantified for the mean amplitudes recorded at electrodes within the parietal (P3, Pz, and P4) regions for P3b.

### 2.6. Experimental procedure

**Figure 1:**
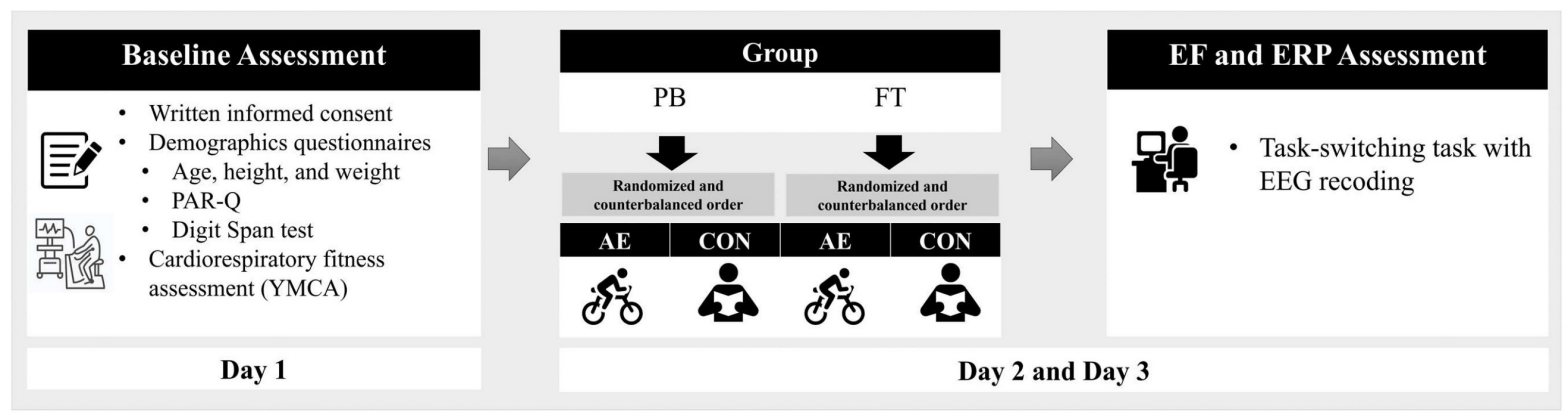
Experimental procedure. *Note.* AE: aerobic exercise session; CON: control session; PB: preterm birth; FT: full-term; EF: executive function; ERP: event-related potential; EEG: electroencephalography.

All participants were invited to visit the laboratory for three days as part of the study protocol (**Figure 1**). During their first visit, the children and their legal guardians were provided with a detailed explanation of the experimental procedures. Written informed consent was obtained from all child participants and their guardians, and the participants completed questionnaires related to demographic data, health history, the PAR-Q, and the Digit Span subtest of the Wechsler test 27. Additionally, participants' cardiorespiratory fitness levels were estimated using the YMCA submaximal protocol. On the second and third visits, participants completed the two intervention sessions (i.e., AE and CON) in a randomly assigned and counterbalanced order. Immediately following the cessation of the intervention, the participants were outfitted with a 32-channel Quick-Cap and escorted to a sound-attenuated room where they performed the task-switching task while task-related EEG was recorded. Children were asked to refrain from engaging in vigorous physical activity and consuming food/drink containing caffeine for at least 6 hours prior to their laboratory visits. The study was conducted following the principles outlined in the Declaration of Helsinki and was approved by the Institutional Review Board of National Taiwan Normal University, Taiwan.

### 2.7. Statistical analysis

Independent *t*-tests were performed to compare the demographic data of the two groups. The behavioral indices (i.e., RT and accuracy) of task-switching performance were separately analyzed using a 2 (Group: preterm vs. full-term) × 2 (Intervention: AE vs. CON) × 2 (Global switch Condition: homogeneous vs. heterogeneous) mixed-design ANOVA and 2 (Group) × 2 (Intervention) × 2 (Local switch Condition: non-switch vs. switch) mixed-design ANOVA. Finally, RT and accuracy for global and local switch costs were analyzed separately using a 2 (Group) × 2 (Intervention) mixed-design.

The neuroelectric measures (i.e., the averaged mean amplitudes for P3b) for global switch and local switch were separately analyzed using a 2 (Group) × 2 (Intervention) × 2 (Global switch Condition) and a 2 (Group) × 2 (Intervention) × 2 (Local switch Condition) design, respectively. For significant interactions and main effects, multiple comparisons with Bonferroni post-hoc analyses were performed. Statistical values are presented following Greenhouse-Geisser correction and the partial eta-square (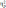) is reported for significant main effects and interactions. All analyses were performed using SPSS (SPSS v.22, Chicago, IL), with a significance level of *p* ≤ .05.

## 3. Results

### 3.1. Participant characteristics

The analyses included data from 20 preterm and 22 full-term children. Independent *t*-tests showed that height, weight, body mass index, and cardiorespiratory fitness levels did not differ between the two groups (*p* > .05 for all comparisons). However, age (*t* (40) = 2.5, *p* < .05), gestational age (*t* (40) = 12.04, *p* < .001), and birth weight (*t* (39) = 13.15, *p* < .001) were significantly different between the groups. The children in the preterm group were older and had a lower gestational age and birth weight than those in the full-term group.

### 3.2. Behavioral measures

#### 3.2.1. Response time

**Figure 2:**
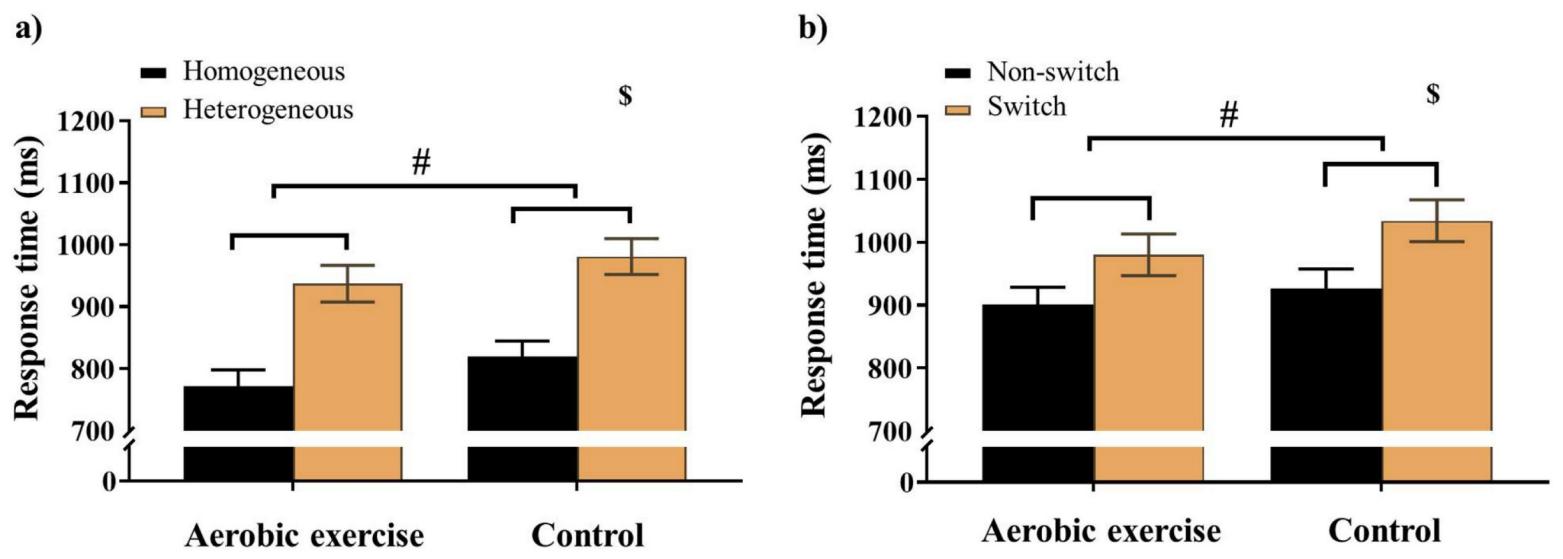
Response time for a) the Global switch; b) the Local switch across aerobic exercise and control interventions. Error bars represent the standard error of the mean. *Note.* $: significant difference between conditions; #: *p* < .05, main effect of Intervention.

For the Global switch condition, a three-way ANOVA revealed a significant main effect of Intervention (*F* (1,38) = 5.10, *p* = .03, 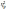
= 0.12), with shorter RTs for AE (853.04 ± 27.37 ms) than for CON (899.29 ±26.17 ms). A significant main effect of Condition (*F* (1,38) = 75.66, *p* < .001, 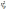
= 0.67) was also observed, with shorter RTs for homogeneous (796.33 ± 24.05 ms) than for heterogeneous trials (956.00 ± 28.54 ms) (**Figure 2a**). No other significant main effects or interactions were found for the RT of the Global switch Condition (*p*s > .05 for all comparisons) (**Table 2**).

For the Local switch Condition, a three-way ANOVA revealed a significant main effect of Intervention (*F* (1,40) = 4.13, *p* ≤ .05, 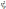
= 0.09), with shorter RTs for AE (941.37 ± 30.04 ms) than for CON (980.86 ± 30.75 ms) (**Table 2**). A significant main effect of Condition (*F* (1,40) = 50.68, *p* < .001, 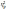
= 0.56) was also observed, with shorter RTs for the non-switch (914.52 ± 27.86 ms) than for switch trials (1007.72 ± 31.12 ms). No other significant main effects or interactions were found for the RT of the Local switch Condition (*p* > .05 for all) (**Figure 2b**).

For the global switch cost and the local switch cost, two-way ANOVA revealed no significant main effects or interactions (*p* > .05 for all comparisons).

#### 3.2.2. Accuracy

**Figure 3:**
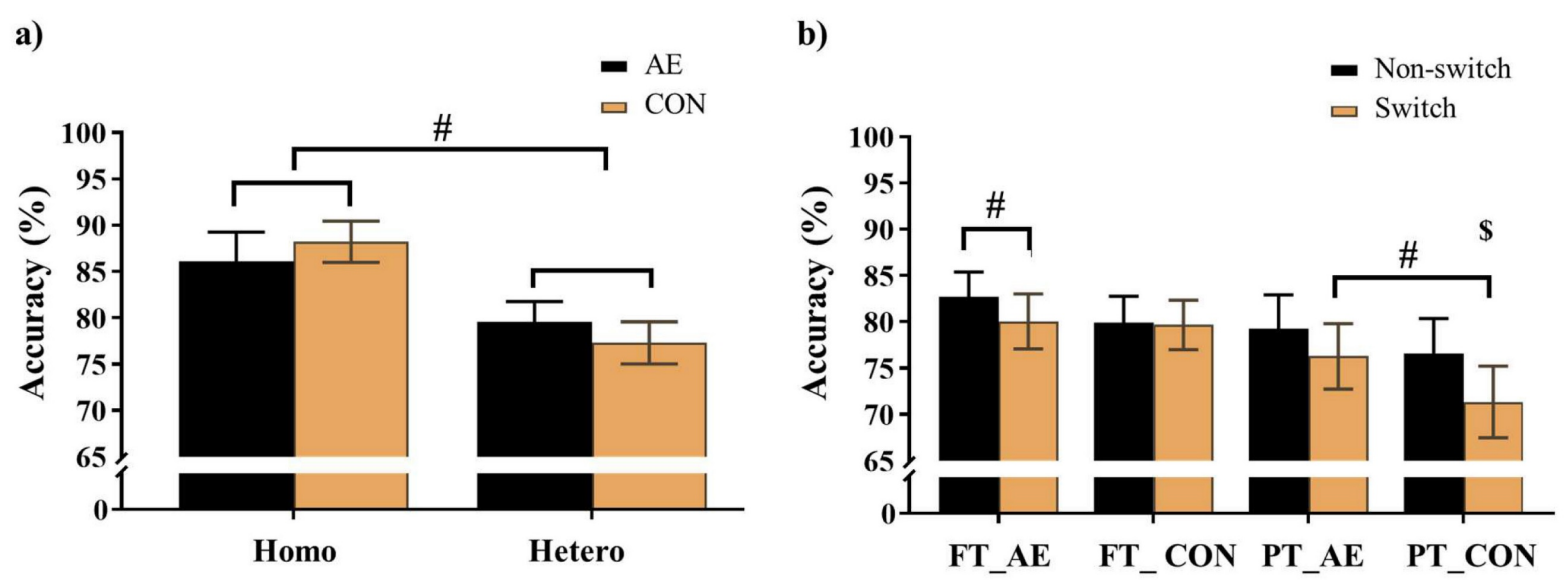
Accuracy for a) the Global switch; b) the Local switch. *Note.* AE: aerobic exercise; CON: control; Hetero: heterogeneous; Homo: homogeneous; FT_AE: full-term aerobic exercise session; FT_CON: full-term control session; PT_AE: preterm aerobic exercise session; PT_CON: preterm control session$: significant difference between non-switch and switch trials for preterm children; #: *p* < .05.

For the Global switch Condition, a three-way ANOVA revealed a significant main effect of Condition (*F* (1,39) = 87.62, *p* < .001, 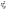
= 0.69), with higher accuracy for the homogeneous trials (88.1 ± 2.1%) than the heterogeneous trials (78.1 ± 2.2%) (**Figure 3a**). No other significant main effects or interactions were found for accuracy (*p*s > .05 for all).

For the Local switch Condition, a three-way ANOVA revealed a significant main effect of Intervention (*F* (1,40) = 4.62, *p* = .04, 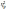
= 0.10), with higher accuracy for AE (79.6 ± 2.2%) than CON (76.9 ± 2.3%). A significant main effect of Condition (*F* (1,40) = 12.75, *p* < .001, 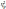
= 0.24) was also observed, with higher accuracy for non-switch trials (79.6 ± 2.2%) than switch trials (76.8 ± 2.2%). The main effects were superseded by a significant Group × Intervention × Local-switch Condition interaction (*F* (1,40) = 6.08, *p* = .02, 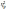
= 0.13). Decomposition revealed that for preterm children, both AE and CON resulted in higher accuracy in non-switch than switch trials (*p*s < .05). Additionally, AE yielded higher accuracy than CON, specifically in switch trials (*p* = .008). For full-term children, only AE resulted in higher accuracy in non-switch than switch trials (*p* = .042), whereas CON showed no significant difference (*p* = .825) (**Figure 3b**). No other significant main effects or interactions were found (*p*s > .05 for all comparisons).

For the global switch cost, the two-way ANOVA revealed no significant main effects or interactions (*p*s > .05 for all comparisons). In contrast, for local switch cost, two-way ANOVA revealed a significant Group × Intervention interaction (*F* (1,40) = 6.08, *p* = .02, 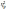
= 0.13). Decomposition of this interaction revealed that the full-term children exhibited significantly smaller local switch cost than the preterm children only during the CON (*p* < .01). No other significant main effects or interactions were found for full-term children of the local switch comparison (*p*s > .05 for all comparisons).

### 3.3. Neuroelectric measures

**Figure 4:**
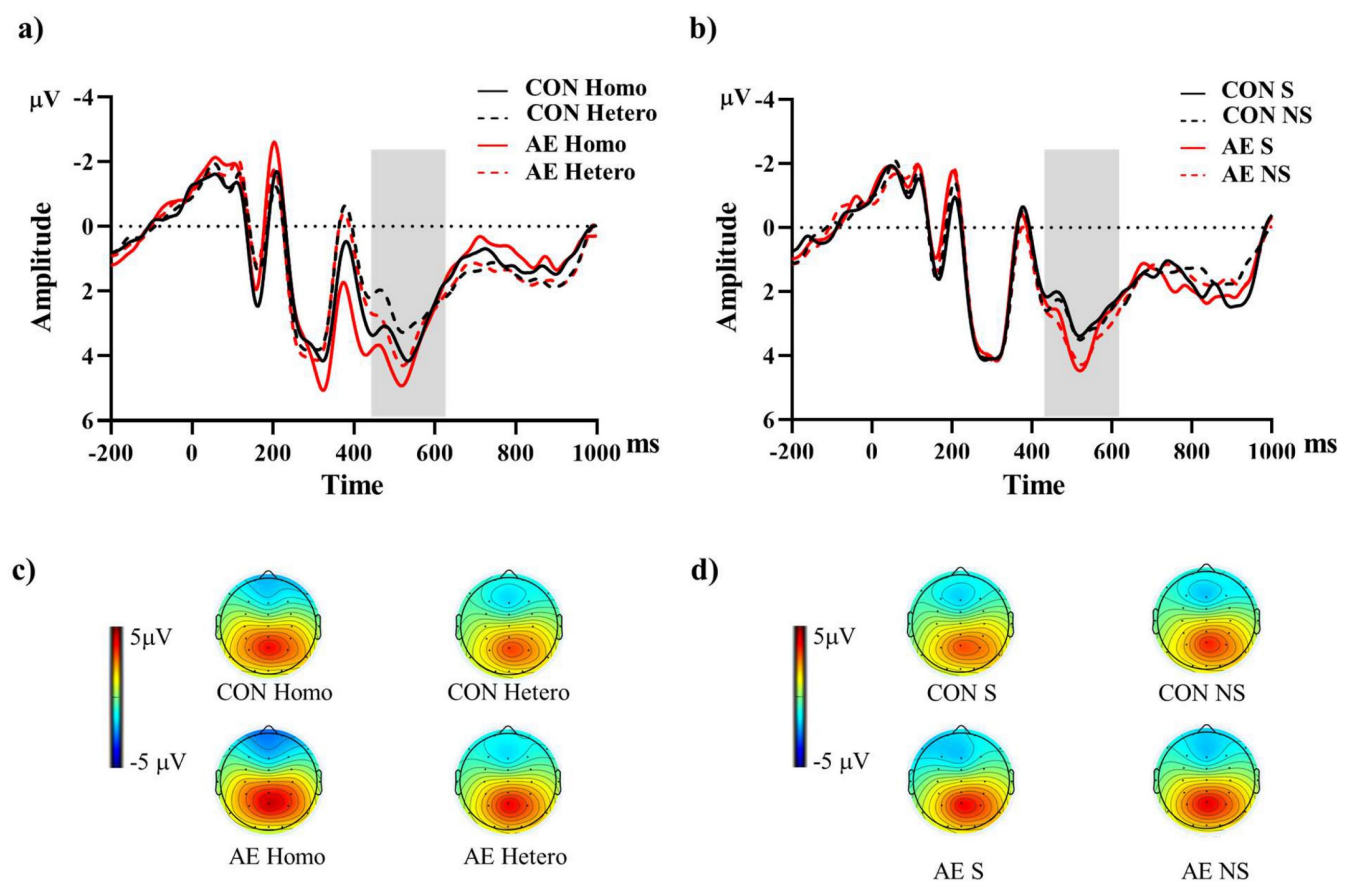
Grand-averaged ERPs from parietal electrodes (P3, Pz, P4) during (a) global and (b) local switch trials under aerobic exercise and control sessions. The shaded interval (430-630 ms) marks the P3b analysis window. Panels (c) and (d) show scalp topographies of mean voltage within this window for global and local switch trials, respectively. *Note.* AE: aerobic exercise; CON: control; Hetero: heterogeneous; Homo: homogeneous; NS: non-switch; S: switch.

The descriptive data for the averaged mean P3b amplitudes of the two groups are summarized in **Table 2**. For the Global switch Condition, a three-way ANOVA revealed a significant main effect of Intervention (*F* (1,34) = 4.63, *p* =.04, 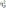
= 0.12), with larger P3b amplitudes for AE (2.54 ± 0.30 μV) than for CON (2.00 ± 0.22 μV) (**Figure 4a and 4c**). Additionally, a significant main effect of Condition was revealed (*F* (1,34) = 7.68, *p* =.01, 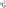
= 1.84), with larger P3b amplitudes for homogeneous (2.50 ± 0.26 μV) than heterogeneous trials (2.05 ± 0.22 μV). No other significant main effects or interactions were found (*p* > .05 for all).

For the Local switch Condition, a three-way ANOVA revealed a significant main effect of Intervention (*F* (1,40) = 4.97, *p* =.03, 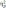
= 0.11), with larger P3b amplitudes for AE (1.87 ± 0.25 μV) than for CON (1.44 ± 0.22 μV) (**Figure 4b and 4d**). No other significant main effects or interactions were found (*p* > .05 for all).

## 4. Discussion

The present study investigated the acute effects of exercise on cognitive flexibility in preterm and full-term children, using both behavioral and neuroelectric measures. Task-switching performance and the P3b component of ERPs were assessed to evaluate EFs in preterm children compared with their full-term peers. The findings showed that children exhibited shorter RTs in both global and local switch conditions and higher accuracy in the local switch condition following a single bout of moderate-intensity aerobic exercise compared with the reading control session. However, switching cost measures did not differ significantly between sessions. In parallel, ERP analyses revealed larger P3b amplitudes after the exercise session across both switching conditions, reflecting greater allocation of attentional resources during task performance. These neural effects, observed in both preterm and full-term groups, suggest that acute exercise may transiently enhance attentional processing rather than cognitive flexibility in the strict sense. Together, these results indicate that preterm children show behavioral and neuroelectric patterns comparable to those of their full-term peers following acute exercise.

Consistent with previous findings in typically developing children 28, the results of the present study suggest that acute aerobic exercise yields modest behavioral benefits for task-switching performance in preterm children. Specifically, children who participated in the acute exercise session exhibited shorter RTs in both Global and Local switch Conditions of the task-switching paradigm, and higher accuracy in the Local switch Condition relative to the CON session. The task-switching paradigm was designed to assess cognitive flexibility, requiring participants to alternate between distinct task rules, inhibit previously relevant responses, and update task representations 29. Our findings align with those of Chen, Yan 30, who reported better performance on an EF task related to cognitive flexibility (i.e., more-odd task), reflected in shorter RTs following a single bout of aerobic exercise. Furthermore, previous evidence indicates that the effects of acute exercise on EF may be more pronounced in children with lower baseline cognitive performance, potentially due to greater room for improvement 31, 32. However, in the present study, switch cost—defined as the performance decrement observed when alternating between tasks compared with repeating the same task, and regarded as a core behavioral index of task switching—was not significantly reduced following the acute exercise session. This finding suggests that certain facets of cognitive flexibility, particularly those involving task-set reconfiguration and response shifting 33, may be less susceptible to the transient influence of a single bout of aerobic exercise.

The present finding that children, regardless of perinatal history, may exhibit comparable cognitive benefits in response to acute aerobic exercise is consistent with prior research demonstrating the benefits of acute exercise on EF in neurodevelopmentally at-risk populations. For instance, Ludyga, Gerber 15 observed that acute exercise facilitated performance on the task-switching task in both children with ADHD and their typically developing peers, indicating that such benefits are not confined to a particular clinical group. Similarly, Hung, Huang 16 reported significantly shorter RTs during task-switching in children with ADHD following a single 30-minute session of moderate-intensity aerobic exercise, suggesting more efficient cognitive flexibility processing. Beyond cognitive flexibility, acute exercise has also been shown to improve other EF domains, such as inhibitory control, in children with ADHD 34-36. Extending this line of evidence, Maltais, Gane 37 also found enhanced inhibitory control, measured via a pediatric Stroop task, in children with cerebral palsy following acute exercise, whereas typically developing children showed positive but limited responses. Taken together, these findings suggest that acute exercise may yield modest benefits across multiple components of EF in diverse neurodevelopmental populations. Although the effects may vary across diverse populations, the present findings support acute exercise as an effective strategy for enhancing EF in children with developmental vulnerabilities, thereby extending prior research on children born preterm 17, 18.

In addition to behavioral improvements, this study provides neuroelectric evidence that participants exhibited increased P3b amplitudes across global and local switch conditions following exercise, irrespective of birth status. Given that P3b amplitude reflects the allocation of top-down attentional resources and is sensitive to cognitive load and task relevance 38, this pattern suggests enhanced efficiency of executive control processes during task execution. These findings align with prior work in typically developing children by Chu, Kramer 39, who observed greater P3b amplitudes following 30 minutes of aerobic exercise during Stroop task performance, interpreted as heightened neural responsiveness and cognitive readiness. Importantly, our study is among the first to demonstrate acute exercise-induced ERP modulations in preterm children, a population characterized by structural and functional alterations in frontoparietal regions critical to P3b generation 40, 41. The larger P3b observed in the present study underscores the retained neuroplastic potential of the brain for preterm children and extends prior evidence from neurodevelopmental populations such as children with ADHD, where similar enhancements in P3b and task-switching performance have been documented following acute exercise 42, 43. While the magnitude and distribution of these effects may vary as a function of developmental diagnosis or birth history 19, the present findings support the generalisability of acute exercise as an approach to engaging attentional control networks in preterm children.

The behavioral and ERP findings suggest that acute aerobic exercise may function as a cognitive primer, temporarily enhancing the efficiency of executive processes in preterm children. Such effects likely arise from increased physiological arousal and catecholamine release, along with transient upregulation of neurotrophic factors such as BDNF, which together facilitate synaptic plasticity and communication among prefrontal, parietal, and hippocampal regions 44-46. Acute exercise may also strengthen large-scale connectivity within the frontoparietal control and attention networks^47^, thereby supporting more effective top-down regulation of behaviour. From a neurophysiological perspective, the ERP modulations are consistent with adaptive gain theory, which posits that transient activation of the locus-coeruleus norepinephrine system optimizes attentional engagement 48 and with neurovascular coupling mechanisms that improve cortical efficiency through enhanced cerebral perfusion and oxygen delivery 49. Given that P3b amplitude and latency are recognized markers of the developmental maturation of executive control networks 50, the similar enhancement observed in both preterm and full-term children suggests that acute exercise may transiently align or normalize attentional processing efficiency in preterm populations.

Several limitations of this study should be acknowledged. First, the relatively small sample size might have limited statistical power and reduced the generalizability of the findings. Larger and more diverse cohorts are needed to validate these preliminary results. Second, the preterm and full-term groups differed in mean age, which may have confounded group comparisons. Future studies should recruit more tightly age-matched samples to minimize developmental variability. Moreover, socioeconomic status and broader cognitive abilities (beyond Digit Span) were not assessed in the present study. We suggest that future research include these variables to provide a more comprehensive understanding of group differences. Third, the study focused exclusively on cognitive flexibility using ERP measures and omitted other EF domains such as inhibition and working memory. A more comprehensive EF assessment would offer a fuller understanding of the cognitive effects of acute exercise. Fourth, alternative explanations for the observed effects should be considered. In particular, motivational, expectancy, or placebo effects, as well as cognitive arousal arising from the novelty or movement inherent in the exercise condition, may have contributed to the performance differences. In addition, uncontrolled factors related to the content or engagement level of the reading control session could also have influenced the results. These possibilities should be addressed in future studies to better isolate the specific contribution of acute exercise. Finally, while this study examined the immediate effects of a single exercise session, future research should investigate the long-term cognitive and neural outcomes of repeated interventions, determine optimal exercise parameters (e.g., type, intensity, and duration), and evaluate whether cognitive improvements translate into real-world functional gains. Complementary neuroimaging modalities such as functional magnetic resonance imaging (fMRI) and functional near-infrared spectroscopy (fNIRS) could further elucidate the neural mechanisms underlying these effects in preterm children.

In conclusion, this study compared the effects of acute aerobic exercise on cognitive flexibility in preterm and full-term children using both behavioral and neuroelectric measures. Behaviorally, children who engaged in the acute exercise session, regardless of birth status, demonstrated shorter RTs in both Global and Local switch Conditions, along with higher accuracy in the local switch condition, compared to the control session. Neuroelectric findings similarly revealed larger P3b amplitudes in both switching conditions following exercise, suggesting enhanced allocation of attentional resources during task performance. Together, these results provide preliminary evidence that a single session of moderate-intensity aerobic exercise can enhance task-switching performance and its underlying neural mechanisms in both preterm and full-term children.

## Figures and Tables

**Table 1:** The demographic characteristics of the two groups (mean ± SD)

	Group	
Variable	Preterm (*n* = 20)	Full-term (*n* = 22)
***Background***		
Age (year)	12.50 ± 1.43	11.59 ± 0.85*
Female (%)	7 (35%)	10 (45%)
Height (cm)	149.15 ± 9.27	147.64 ± 8.81
Weight (kg)	43.00 ± 8.37	39.59 ± 12.20
BMI (kgm^-2^)	19.33 ± 3.58	17.86 ± 3.82
VO_2max_ (mL.kg^-1^.min^-1^)	40.17 ± 10.1	44.58 ± 18.72
***Perinatal history***		
Gestational age (week)	29.80 ± 3.32	38.68 ± 0.95*
Birth Weight (g)	1273.16 ± 469.78	3170.14 ± 452.51*
***Digit span***		
Forward	13.95 ± 1.91	12.24 ± 2.49
Backward	7.30 ± 2.90	7.29 ± 2.17

*Note.* BMI: body mass index; SD: standard deviation; VO_2max:_ maximum volume of oxygen consumed per unit time; *:* p* < .05.

**Table 2:** Behavioral and neuroelectric indices for group and intervention (mean ± SE).

Measures	Group			
	Preterm (*n* = 20)		Full-term (*n* = 22)	
	AE	CON	AE	CON
**RT (ms)**				
Homo	794.21 ± 39.18	843.32 ± 38.48	753.96 ± 36.67	793.82 ± 36.15
Hetero	946.08 ± 44.94	983.11 ± 43.40	917.90 ± 42.41	976.91 ± 50.42
NS	913.54 ± 39.81	944.36 ± 45.24	890.10 ± 37.96	910.07 ± 43.14
S	1003.15 ± 48.12	1046.92 ± 48.68	958.71 ± 45.88	1022.11 ± 46.41
GSC	151.87 ± 28.58	139.79 ± 31.24	163.93 ± 27.65	183.09 ± 42.61
LSC	89.50 ± 16.01	110.09 ± 30.87	68.62 ± 14.88	112. 04 ± 28.69
**ACC (%)**				
Homo	87.3 ± 3.5	87.7 ± 3.3	89.0 ± 3.4	88.2 ± 3.2
Hetero	77.8 ± 3.2	74.5 ± 3.3	80.7 ± 3.1	79.5 ± 3.2
NS	79.3 ± 3.2	76.6 ± 3.4	82.7 ± 3.1	79.9 ± 3.2
S	76.3 ± 3.3	71.3 ± 3.4	80.0 ± 3.2	79.7 ± 3.2
GSC	9.6 ± 1.9	13.1 ± 2.0	8.3 ± 1.9	8.7 ± 2.0
LSC	3.0 ± 1.3	5.2 ± 1.3	2.7 ±1.3	0.3 ± 1.2
**P3b (μV)**				
Homo	2.85 ± 0.51	2.44 ± 0.38	2.67 ± 0.46	2.02 ± 0.34
Hetero	2.17 ± 0.42	1.65 ± 0.34	2.48 ± 0.38	1.88 ± 0.31
NS	1.76 ± 0.38	1.37 ± 0.35	1.97 ± 0.36	1.68 ± 0.33
S	1.80 ± 0.36	1.23 ± 0.31	1.94 ± 0.34	1.49 ± 0.29

*Note*. ACC: accuracy; Hetero: heterogeneous; Homo: homogeneous trials; GSC: global switch cost; LSC: local switch cost; NS: non-switch; RT: response time; S: switch; SE: standard error; AE: aerobic exercise session; CON: control session
